# What makes patients in primary care complex? A scoping review combined with a focus group analysis

**DOI:** 10.1080/02813432.2025.2507278

**Published:** 2025-05-17

**Authors:** Jozé Braspenning, Rachel van den Kieboom, Erik W.M.A. Bischoff, Kirsten Hietland, Kris Vissers, Yvonne Schoon

**Affiliations:** ^a^ Centre of Integrated Care, Radboud University Medical Center, Nijmegen, the Netherlands; ^b^ Radboud Institute for Health Sciences, Scientific Centre for Quality of Healthcare (IQ Healthcare), Radboud University Medical Center, Nijmegen, the Netherlands; ^c^ Radboud Institute for Health Sciences, Department of Primary and Community Care, Radboud University Medical Center, Nijmegen, the Netherlands; ^d^ Department of General Practice, Erasmus MC University Medical Center, Rotterdam, the Netherlands

**Keywords:** Primary care, patient complexity, integrated care, multimorbidity, quality of care, good health and well-being

## Abstract

**Background:**

Consultations can be challenging because of patient complexity, affecting care quality. Patient complexity involves factors related to the patient, healthcare professional, organization, and healthcare system. A comprehensive overview of these factors and their significance to healthcare providers is lacking. The aim of this study was to create a literature overview and to examine how the results relate to the experiences of primary care providers.

**Methods:**

The PubMed, Embase and Cochrane databases were searched (2018–2023) to conduct a scoping review. In addition, a focus group was organized with healthcare providers from general practices (a) to discuss the results of the review, and (b) to explore its meaning in daily consultation, based on the real-life patient cases they presented.

**Results:**

The review (171 unique studies) mainly revealed patient-related factors (medical, social and behavioural aspects). Complexity arises when multiple aspects are present and mutually interact. Factors related to the expertise of healthcare professionals, the organization (care coordination) and the system (availability of resources, navigation problems) were also identified. The focus group recognized the factors that influenced patient complexity and their interdependence. They noted a need for support in identifying and treating the patient population in question across disciplines.

**Conclusions:**

A comprehensive overview of factors influencing patient complexity at four different levels (patient, professional, organization, system) is provided. The primary care providers suggested disseminating these results to customize treatment to the needs of patients, which is likely to require coordination across disciplines and integrated care.

## Introduction

In primary care, patient complexity poses a challenge in daily consultation [1], and this affects the quality of care [2]. Patient complexity is associated with multimorbidity (i.e. patients with two or more chronic conditions) [3]. Over time, the definition of patient complexity has been expanded to include other influencing factors. It refers to patients facing significant challenges in terms of both medical and nonmedical aspects (e.g. social well-being, income, housing, health literacy) [4–6]. These influencing factors are all at the level of the patient. To better meet the needs of these patients, algorithms have been developed based on medical records data to improve the detection of risk factors related to patient complexity [7]. The codes used in these medical records are medical, which limits the signalling function of such algorithms. In addition, various scholars and practitioners have used a variety of complexity-related concepts to design tools or questionnaires to identify patient complexity [8]. The patients identified using these tools differ according to the definitions of patient complexity used. Unfortunately, a clear definition of factors related to multidimensional patient complexity is currently lacking.

Healthcare policy research shows that, beyond patient-related factors, managing patient complexity depends on factors related to healthcare professionals, organizations, and systems [9–11]. For example, complexity may be related to a lack of multidisciplinary professional guidelines (healthcare professional), care coordination (organization) or healthcare financing (system). To date, however, no comprehensive overview of these factors has been prepared. Such an overview could support healthcare providers in their efforts to address patient complexity in order to improve the quality of care.

The aim of this study was to conduct a literature review and a focus group with primary care providers to identify what makes patients in primary care complex. The study was designed from the perspective of the primary care provider in a general practice. This choice was made because, in the Dutch healthcare system, the management of patient complexity is entrusted to general practitioners, given their gatekeeping function and role in delivering continuity of care [12,13].

## Methods

Multiple data source methods were used to gain a more comprehensive understanding of patient complexity. First, we conducted a scoping review to investigate the scope and content of the literature on patient complexity [14]. Second, a focus group was held with primary care providers (PCPs) to explore primary care’s awareness of this knowledge and its impact on daily consultations. Both activities are described separately below.

### Scoping review

#### Search strategy, data extraction and analysis

A scoping review was conducted according to the PRISMA-ScR guidelines [15]. PubMed, Cochran and Embase were searched for English-language articles published in the period January 2018–February 2023, reporting on factors that influence patient complexity in adults. We limited our search to the last five years, given a recent publication of a comprehensive review (83 studies) on patient complexity [16]. The search string is presented in Appendix 1. Articles describing only clinical factors (e.g. complicated surgery) or a single specific condition (e.g. rheumatoid arthritis) or a measurement tool were excluded. The data were downloaded and screened by using Rayyan [17]. Two authors (RK, KH) read the articles and extracted relevant data, including first author, year of publication, type of article, setting and factors related to patient complexity. The references of the articles included were screened to identify additional relevant articles. The factors were clustered into patient, healthcare professional, organization and system-related aspects. Discrepancies or ambiguities were resolved in consultation with a senior researcher (JB).

### Focus group

#### Study design and participants

A focus group was organized to learn more about the patient complexity experiences of primary care providers, i.e. general practitioners (GP) and practice nurses. Participants were recruited through convenience sampling in general practices in the eastern region of the Netherlands. General practices that agreed to take part were sent an invitation to join a digital focus group and requested to share a complex patient case. Four general practitioners and a practice nurse took part. Two of these participants were working in a small municipality, one was working in a medium-sized municipality, and two were working in a large municipality. The participants ranged in age from 30 to 50 years, and four of the five were women. The GPs had between 7 and 21 years of experience, and the practice nurse had one year of experience. EB, one of the co-authors, was a focus group participant. To prevent bias, he was kept unaware of the scoping review’s design, results, and the method of the focus group beforehand.

#### Data collection

The focus group guide included three topics: (1) exploration of the concept of patient complexity using a ‘Wordle’; (2) reflection on the factors of patient complexity, as identified in the scoping review; and (3) discussion of the submitted patient narratives to gain insight into the experiences of PCPs with patient complexity in daily consultations. The focus group questions from the topic guide are presented in Appendix 2. The focus group was moderated by an experienced moderator (JB), and two researchers (RK, KH) were present to observe, take notes, and ask additional questions. At the end of the focus group, the moderator summarized the discussion and invited the participants to reflect on the validity, saturation, and accuracy of the information. The focus group (105 min) was conducted through Microsoft Teams, which generated automatic video and audio recordings and transcription. After two weeks, participants received a summary of the focus-group meeting by email, and they were given the opportunity to provide feedback. No feedback was provided. As compensation for participation, the GPs received two accreditation points, and the nurse received a gift voucher valued at €25.

#### Data analysis

The automatically generated transcript was checked and corrected by the researchers (RK, KH) based on the video and audio recordings. A deductive content analysis [18] of the focus group transcripts was conducted by the two researchers (RK, KH) separately, using ATLAS.ti 23.0.7. We categorized factors as patient, healthcare professional, organizational, or system-related to combine the focus group analysis and scoping review. Any disagreements were discussed with a third researcher (JB). The results of the focus group were discussed in weekly meetings, during which was decided that data saturation was achieved. The focus group is reported according to the COREQ criteria for qualitative studies [19].

## Results

### Scoping review of patient complexity

#### Review characteristics

**Figure 1.**
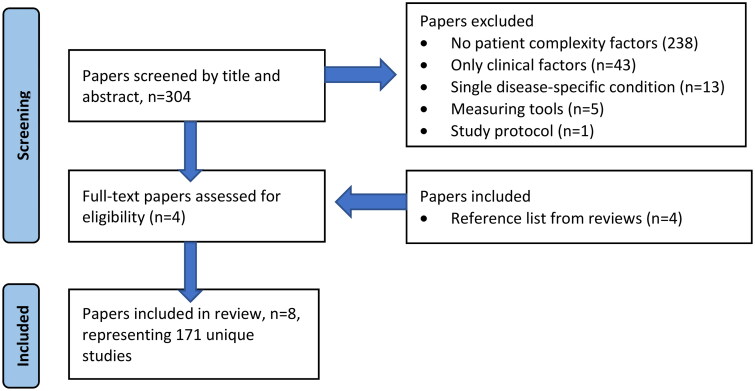
Flow chart literature review.

Eight articles were included in the scoping review. The search in PubMed, Cochrane and Embase yielded 304 unique articles, four of which [16,20–22] were included, based on the inclusion criteria. Among the articles was a wide-ranging review encompassing 83 studies [16]. We separately reviewed four of the 83 studies that met our inclusion criteria, despite a publication date outside our search period [5,23–26], because details could have been lost in such a large review. The details of these studies are described in the review. An overview of the articles selected for the scoping review is provided in Figure 1.

The characteristics of the articles included are listed in Table 1.

**Table 1.** Characteristics of the articles included.

First author	Year of publication	Design	Sample	Setting
Nicolaus [16]	2022	Narrative review	83 studies	Outpatients, adults
Grant [23]	2011	Cohort study with a case review	40 primary care physicians	Primary care
Loeb [24]	2015	Qualitative in-depth interviews	15 healthcare professionals	Primary care
Mount [25]	2015	Action research with a case description	13 primary care physicians	Primary care
Schaink [26]	2012	Scoping review	127 studies	Outpatients, adults
Ben Menahem [20]	2021	Semistructured in-depth interviews	31 healthcare professionals	Outpatients and patients with HIV
Strachan [21]	2023	Modified Delphi study	28 healthcare professionals	Outpatients, adults with psychological problems
Webster [22]	2019	Qualitative institutional ethnography with interviews	51 primary care professionals	Primary care

#### Factors related to patient complexity

The scoping review revealed a variety of factors related to patient complexity. Table 2 provides an overview by categorizing the factors in a framework that distinguishes between factors related to the patient, healthcare professionals, organization and healthcare system. Patient-related factors appeared in all articles included [5,16,20–25] and could be categorized as medical, social or health behaviour-related factors. The presence of medical factors (e.g. multimorbidity, polypharmacy, psychiatric disorders and psychological vulnerability) can increase patient complexity, as can limited cognitive capacity. Social factors related to demographics (age, gender, disparities, culture, socio-economic status (SES)) are more common amongst women, older people and people with low SES (associated with disparities and migration background). Most studies [16,20–24] report barriers related to health literacy and language separate from demographics. Such factors can contribute to patient complexity. Social capital can help reduce patient complexity, whereas challenging life circumstances can increase patient complexity. Factors related to health behaviour were divided according to four aspects: coping, passive approach to healthcare, patient-doctor relationship, and secondary gain. Ability to cope with crises, circumstances and demands can reduce patient complexity, as can a good patient-physician relationship. A passive approach to healthcare, and the secondary gains from appearing complex, can also increase patient complexity. Healthcare professional factors, including experience, expertise, strong doctor-patient relationships, and effective interprofessional communication, all correlated positively with patient complexity. Organizational factors concerned the ‘coordination of multiple providers and services’. A lack of clear agreements and coordination can contribute to patient complexity. Finally, the following system-related factors were described: ‘patient access’, ‘resources’ and ‘navigation’. Patient complexity was linked to insufficient knowledge and understanding of these factors. The key message of all articles was that factors are often interrelated, both between and within categories. It was also concluded that a specific combination of factors applies to each patient. Some factors are a product of a combination of several factors, including ‘frailty’ [1,16,25], ‘chronic pain’ [22,23], ‘heavy healthcare use’ [5,16,24,25] and ‘poor quality of life’ [25]. Some factors (e.g. ‘sudden instability’ or ‘unexpected event’) have been described as triggers for identifying complexity [16,20,24,25].

**Table 2.** Patient complexity factors classified from the findings of the scoping review.

Level	Domain	Factors	References
Patient	*Medical*	**Multimorbidity** (chronic)	[5,16,20–22,24–26]
		**Severity, risk**	[16,21,26]
		**Polypharmacy:** interactions, side effects	[16,20–22,26]
		**Psychiatric disorders:** personality, anxiety, depression, substance abuse addiction	[16,20–22,24,26]
		**Psychological vulnerability**	[5,16,20–22,24–26]
		**Cognitive capacity**	[16,21,24–26]
	*Social*	**Demographics:** age, gender, (ethnic) disparities, culture, SES (education, income, job)	[5,16,20–22,24–26]
		**Health literacy,** language barriers	[16,20–22,24,25]
		**Social capital**: network, informal caregiver strain	[16,20–22,25,26]
		**Challenging life circumstances**: family stressors, lack of social safety, housing	[5,16,20–22,24–26]
	*Health behaviour*	**Coping**: burden of disease, resilience	[16,20–22]
		**Passive approach to healthcare**: treatment adherence, self-management, missed appointments	[16,20,22,25,26]
		**Patient-doctor relationship**	[16,21,22,25]
		**Secondary gain**	[21]
Healthcare professional		**Experiences and expertise in patient care**	[16,20,21,24,26]
		**Patient-doctor relationship**	[16,21,26]
		**Interprofessional and interdisciplinary communication**	[16,20,21,26]
Organization		**Coordination of multiple providers and services**	[16,20–5]
System		**Patient access**	[21,22]
		**Resources for healthcare professionals (e.g. time)**	[16,20,22]
		**Difficulty navigating the healthcare system**	[22,25,26]

### Focus group on patient complexity

Focus group results confirmed the scoping review’s findings on factors influencing patient complexity. The discussion based on the patient narratives emphasized that patient complexity varies widely across individual patients. The new framework, in which patient complexity not only relates to patient factors but also to characteristics of healthcare professionals, the organization of care and the care system, provided a grip on describing daily practice.

### Patient factors

The PCPs in the focus group recognized that complex patients often have ‘multiple medical problems’, which can be manifested at both the somatic and the psychological level. It was discussed that psychological factors may be presented as somatic symptoms. Such somatization was a frequently observed factor in complex patients, leading to an increased number of consultations. The participants pointed out that the management of unrelated physical symptoms can pose a challenge to making progress in treatment, and because of that contributing to patient complexity.

*… that you recognize physical complaints, but have the idea that they are really arising from a psychiatric disorder.[P04] … it can also be a psychological manifestation: a physical manifestation or psychological dysregulation.[P02]*

As noted by some PCPs, the treatment of complex patients should focus more on psychosocial aspects than on the somatic component. They further observed that, even though complex patients often come to consultation with somatic complaints, it is also important to address the related psychosocial component, which is often hidden.

*I think that is a genuine breakthrough; the patient no longer focuses only on the somatic part but also on the psychosocial part. However, as long as she (the patient) is not open-minded, you need another entrance into the conversation to discuss the problem. [P04]*

Participants also suggested that the complexity of a patient is often related to issues in the ‘social domain’, which are sometimes prominent and sometimes latent.

*It’s very clear to me that social components play a significant role, don’t they? Loneliness, having support, lack of social network. [P04]*

As also emphasized by the PCPs, however, complexity varies from one individual to another, and each case is unique. They shared that complexity is not determined by the specific circumstances of an individual case, but by a combination of factors.

Participants further noted that complexity is influenced by an individual’s response to and ability to cope with these circumstances. They recognized ‘patient behaviour’ as an area that contributed to patient complexity, and they identified several subcategories within this domain, including medication adherence and limited health literacy.

Sometimes, you see indifference in people, don’t you? Leave things as they are. For example, ‘I can’t keep track anymore’. This endangers treatment compliance. Alternatively, even despite all explanation and support, some people truly do not have sufficient health skills to keep track. [P04]

Patient coping strategies comprised another identified behavioural factor. Patients may have difficulty with coping strategies because of an inability to cope, as well as because they may not be open to accepting certain advice or shifting the focus from the somatic component.

She (the complex patient in the case) was so incredibly fixated on the somatic part. She was not open to accepting her symptoms and learning to deal with her pain. … but, for this woman, it (having a holistic vision) just doesn’t work, because she doesn’t seem to be open to it herself. [P02]

### Factors related to healthcare professionals

According to the PCPs, factors related to healthcare professionals had both a positive and a negative influence on patient complexity. One important factor discussed was ‘having experience’ working with complex cases (personal competence). Primary care providers were unsure how to address or refer patients with complex needs.

Maybe the patient whose… Yeah… It’s not clear, yeah…. When you don’t have a clue, when you think, ‘What’s going on here? What’s behind all of this?’ [P05]

The PCPs highlighted a training emphasis on medical health, limiting their exploration of patients’ diverse needs and expectations within the context of complex cases. They proposed that a shift towards a more holistic patient approach would be desirable to better meet the needs of complex patients.

Well, I don’t think that’s (social domain factors) something that we learned in our training, because we were taught to make people better. There also comes a point at which health is no longer the most important thing, but well-being is. I think that this is not routine for many doctors…. However, it’s actually a shift that we have to make in our thinking pattern. [P01]

The PCPs discussed the fact that patient complexity was also related to the ‘patient-physician relationship’. In the Dutch primary-care system, a patient can be registered in only one general practice, and the healthcare professionals in that practice are quite familiar with the condition and context of that specific patient. PCP consensus: while this setting aids care continuity, established relational patterns may reduce patient complexity. Having an additional healthcare professional with expertise in complex patients, employing specialized diagnostic methods and varied viewpoints, might be advantageous.

However, I think sometimes it works better if a more objective person can have a conversation with her (a complex patient) and see if that can motivate her in a certain direction. [P02]

Finally, the importance of ‘interprofessional communication’ was discussed. The involvement of multiple healthcare providers in the management of complex patients may lead to various problems for PCPs. The lack of communication between healthcare professionals creates unclear directions and a lack of overviews. Limited interprofessional communication and coordination between responsible healthcare professionals can increase misunderstanding of the complexity of an individual patient. Organizational factors might promote interprofessional communication.

### Organizational factors

The PCPs recognized that the organization of care can also influence patient complexity. They noted that healthcare professionals seem to approach patients primarily from their own personalities and areas of expertise, instead of first trying to understand the needs and expectations of the patients, thus hindering the coordination of care.

Complexity may not even be the somatic or medical suffering itself, but also the multitude of specialists involved… all the advice and medications and therapies that result from it that conflict with each other, or overlap and have no connection. In addition, a multitude of healthcare providers are involved. This sometimes means I, as a GP, lose control, leaving older patients and their informal caregivers without a clear understanding of how things can work together effectively. [P04]

The PCPs who were interviewed indicated that better interprofessional coordination between the various disciplines involved could improve the organization based on the needs and expectations of the patient.

### System factors

Participants discussed the situation that ‘patient access’ is at stake because of a lack of availability of healthcare services and waiting lists. The PCPs agreed that this occurs in various settings. Mental health and hospital care were used as examples.

Especially now, when people don’t have access to mental healthcare or hospital services because of a six-month waiting period. [P03]

The PCPs also reported that responding to patient complexity requires more time, which is challenging because of staff shortages and insufficient reimbursement (‘resource problems’). In addition, several participants mentioned ‘difficulties navigating’ the healthcare system in finding the most appropriate solution, which affected both patients and healthcare professionals. It is difficult to find one’s way in a complex healthcare system characterized by constant changes in healthcare and organization.

Overall, PCPs did not envision any simple measurement tool that could identify patient complexity, as the concept is too multidimensional and multifactorial, as well as a wide variation in the influencing factors among patients. The PCPs recommended spreading knowledge about the various factors associated with patient complexity and how they might be interrelated and unique for each patient.

## Discussion

### Main findings

Our scoping review shows that patient complexity involves various factors related to patients, healthcare providers, the organization, and the healthcare system. The results presented in a new framework showed that patient complexity is a multifaceted concept, and many influencing factors can interact and differ among patients. The descriptions that primary care providers (PCPs) in general practice gave concerning their experiences with patient complexity overlapped with the factors identified in the scoping review, and this correspondence was not limited to patient-related factors. Professional expertise, the organization and the health care system related to their readiness of managing the complexity. The argument was made that identifying the target group could improve quality of care. The primary care providers had no great expectations for the use of dedicated measuring tools to identify patient complexity. They called for the dissemination of knowledge among their colleagues concerning the influence of these factors on complexity to improve the management of care based on the needs of individual patients.

### Strengths and weaknesses

One strength of this study is its use of a combination of the two different research methods.

The various factors from the scoping review were given more practical meaning for primary care by presenting and discussing several patient cases in the focus group (data triangulation). Another strength was the separate inclusion of some studies from the review [16], which provided additional information. Despite these strengths, the study is also subject to several limitations related to the focus-group method. Although it is often advised to organize at least two focus groups [26] to achieve data saturation, the current study is based on only one focus group. Another focus group could have enriched the examples given by PCPs, especially from the perspective of the practice nurse, who was now represented by only one participant. However, we do not expect that the number of factors related to patient complexity would have substantially increased, as the large overlap in findings between the scoping review and the focus group suggests data saturation. The organizational and system-related factors received slightly less attention in the focus group, probably because these aspects were beyond the control of the healthcare professionals. It is also important to note that the data were collected in the Netherlands, and consequently our findings cannot apply automatically to other countries.

### Comparison with the literature

As suggested by the scoping review, patient complexity is a multifaceted concept with influencing factors related to patients, healthcare professionals, care organizations and the healthcare system. We categorized the patient-related factors into three domains: medical, social and health behaviour. The studies from the scoping review [5,16,20–25] used broadly similar terms to generate an overview of the factors influencing patient complexity. The overview of influencing factors is now more complete and the grouping of the different factors can contribute to more clarity for daily practice. From the outset, participants in the focus group explicitly stated that complexity was also influenced by non-patient-related factors. For example, they discussed the appropriateness of the skills of PCPs. Although the demanding nature of patient complexity [1] was not reflected in the results of the scoping review, the participants in the focus group discussed aspects that illustrate this demanding character, including the increased number of consultations, progress in treatment and the search for treatment options.

### Implications for practice and research

A detailed description of patient complexity can facilitate understanding concerning the concept of complexity and provide guidance to primary care providers in delivering high quality of care to this specific population. The focus group emphasized the need for better dissemination through embedding these findings in the educational programmes of the PCP training institutes. Another challenge in the management of patient complexity is coordination across disciplines and professions. As noted by the PCPs in this study, healthcare professionals act primarily within the framework of their own specialities, and this hinders the coordination of care from the perspective of patients. The results of our study may support models of integrated care that have emerged to improve the understanding and coordination of patient complexity [12,27–29]. A concern is that currently no practical selection tool is available within the context of general practice to determine which patients should be included in an integrated care programme, as the tools available address only some factors that influence patient complexity [7,8]. A suggestion is to investigate whether asked a ‘surprise question’ can be a tool to include the eligible population. For example, in palliative care, this ‘surprise question’—’Would I be surprised if this patient were to die within the next 12 months?’—is asked to predict the need for palliative care [30]. The exact wording of the surprise question to recognize patient complexity could likely be related to the various factors identified in this study.

## Conclusions

Results from a scoping review and a focus group with primary care providers clearly suggest that patient complexity is related to many factors at the levels of care, organizations, and systems. In addition, every patient may have a distinct combination of these interacting elements. The primary care providers involved in this study argued that an overview of these factors could improve the ability to customize treatment to the needs of patients, which is likely to require coordination across disciplines.

## Supplementary Material

PRISMA ScR Checklist Patient complexity Scoping review.docx

## Data Availability

The datasets generated and analyzed during the current study are not publicly available, as the informed consent was obtained only for the use by the research team. If desired, the data can be viewed and reviewed together with the corresponding author.

## Appendix 1: Search string scoping review patient complexity

The following search string was applied to the title and abstract: Patient complex* or Complex Chronic Patient in combination with assess*, define*, scor*, model*, cluster*, define, identify*, classif*, typology, framework*, clarif*, concept*, characteriz*, explain, explanation, determin*, explicat*, terminology, screening, conceptualiza*. Truncation (*) was used to capture variations in spelling conventions or hyphenation. Appropriate MESH terms were not available.

## Appendix 2: Focus group questions from the topic guide

1. After introducing the project and the participants, we asked, ‘What aspects do you consider regarding patient complexity?’ The answers were noted in a Wordle, after which clarification was requested by which the discussion started.
2. The results from the scoping review were presented by the moderator according to the categorization of Table 2, and we asked, ‘Do you recognize the factors mentioned in the literature? Do you have any additions?’ and ‘How does patient complexity manifest itself in daily consultation?’
3. The patient cases on patient complexity that the practices had submitted in advance were discussed, and we asked, ‘Would you like to elucidate your case and discuss which factors make the case complex for you?’ This section ended with the following question: ‘What might help you to identify patient complexity in daily practice?’
